# How might bacteriophages shape biological invasions?

**DOI:** 10.1128/mbio.01886-23

**Published:** 2023-10-09

**Authors:** Jannick Van Cauwenberghe, Ellen L. Simms

**Affiliations:** 1 Institute of Biodiversity, Faculty of Biological Sciences, Cluster of Excellence Balance of the Microverse, Friedrich Schiller University Jena, Jena, Germany; 2 Department of Integrative Biology, University of California, Berkeley, California, USA; The Ohio State University, Columbus, Ohio, USA; The Ohio State University, Columbus, Ohio, USA

**Keywords:** bacteriophages, microbial communities, rhizosphere-inhabiting microbes, invasion ecology, multi-trophic interactions, plant-microbe interactions

## Abstract

Invasions by eukaryotes dependent on environmentally acquired bacterial mutualists are often limited by the ability of bacterial partners to survive and establish free-living populations. Focusing on the model legume-rhizobium mutualism, we apply invasion biology hypotheses to explain how bacteriophages can impact the competitiveness of introduced bacterial mutualists. Predicting how phage-bacteria interactions affect invading eukaryotic hosts requires knowing the eco-evolutionary constraints of introduced and native microbial communities, as well as their differences in abundance and diversity. By synthesizing research from invasion biology, as well as bacterial, viral, and community ecology, we create a conceptual framework for understanding and predicting how phages can affect biological invasions through their effects on bacterial mutualists.

## MUTUALIST AVAILABILITY DRIVES HOST INVASIONS

Biological invasion is a fascinating and troublesome phenomenon: it causes major ecological and economic costs but also provides important ecological insights (1 – 3). An invasion occurs when a species introduced to a new range proliferates there and becomes pestiferous (4 – 6). The ability to invade is strongly influenced by the biota, either native or introduced, with which an introduced species interacts in the new range (7, 8).

**FIG 1:**
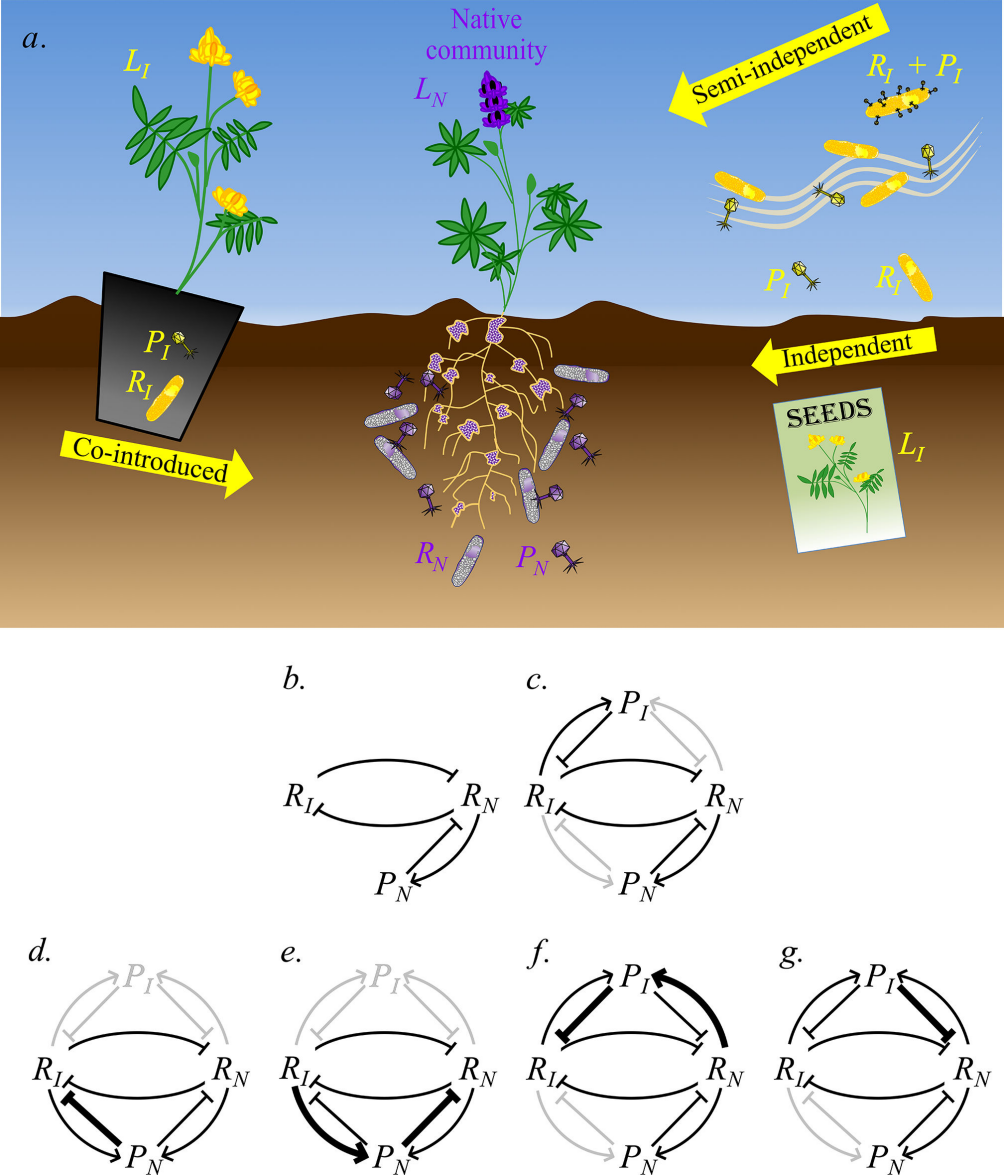
(a) This cartoon depicts different paths along which an invaded community might assemble. In the center, the native community, depicted in cool colors, includes the native legume (*L_N_*), nodulated by its native rhizobium symbiont (*R_N_*), which also lives free in the soil and hosts native phage (*P_N_*). Biological entities from a hypothetical distant home range community, depicted in warm colors, can arrive via several pathways. On the left, a potted legume (*L_I_*) is co-introduced with rhizobium (*R_I_*) and phage (*P_I_*) from its home range. On the lower right, the introduced legume (*L_I_*) arrives as seed, with neither symbiont nor phage. On the top right, rhizobium from the distant home range is co-introduced with phage (*R_I_ + P_I_*). Alternatively, in the center right, phage (*P_I_*) and/or rhizobium (*R_I_*) arrive independently. (b–g) When introduced rhizobia (*R_I_*) and native rhizobia (*R_N_*) compete, the various ways they might interact with phages lead to different invasion biology hypotheses, as described below. (b) Introduced rhizobia (*R_I_*) arrive without their familiar home range phages (*P_I_*), are not infected by native phages (*P_N_*), and compete with native rhizobia (*R_N_*) (“enemy escape”). (c) Both rhizobia and phages are introduced from the same distant population. If each rhizobium is preyed upon only by its familiar phage (gray lines absent), then each rhizobium might be regulated by its familiar phage; however, the relative magnitudes of the dark lines determine if invasion can occur. If either rhizobium is attacked by an unfamiliar phage, the gray lines between that pair are present (dark). Again, several outcomes are possible, as described below. (d) Native phages affect introduced rhizobia more negatively than native rhizobia (“biotic resistance” via “reverse spillover”). (e) Native phages are strongly amplified by introduced rhizobia but more negatively affect native rhizobia (“enemy spillback”). (f) Introduced phages are strongly amplified by native rhizobia but more negatively affect introduced rhizobia (biotic resistance via “subsidized pathogen”). (g) Introduced phages more negatively affect native rhizobia than introduced rhizobia (“enemy spillover”). Note that panels d and e can occur, regardless of the presence of introduced phages, and that panels f and g can occur, regardless of whether native phages can attack introduced rhizobia. Lines indicate the direction of the interaction effect. Arrowheads indicate interactions that increase fitness of the biological entity at which the arrowheads point, while a flathead indicates interactions that decrease fitness of the receiving partner. Interactions and partners depicted in gray might be either present or absent, as described above. Arrow thickness indicates the magnitude of the interaction effect.

Many eukaryotes depend on bacterial mutualists that are horizontally transmitted [Table 1 (9 – 13)]. These bacteria do not disperse with host propagules and instead infect the host from a free-living stage. Thus, for mutualist-dependent eukaryotes to invade, these horizontally transmitted symbionts must arrive independently and survive as free-living bacteria (4, 14 – 19). Bacteriophages, viruses that infect bacteria, strongly shape bacterial community composition (20 – 29). Thus, as a eukaryotic mutualist moves into a new range, its fate could hinge on the ecological and evolutionary outcomes of bacterium-bacteriophage interactions encountered there by the free-living bacterial symbionts on which it depends. Here, we explore how rhizobiophages, bacteriophages that specialize on rhizobia, could affect range expansions by legumes and rhizobia (Fig. 1), which is a well-studied model of mutualist-dependent invasion. To do so, we introduce invasion biology and then use it to predict how phages could influence an invasion by a mutualist-dependent eukaryote.

**TABLE 1:** Examples of environmentally acquired mutualistic bacteria that associate with invasive eukaryotic hosts

Invasive eukaryote	Mutualistic bacterium	References
*Alnus glutinosa* (European alder)	*Frankia* sp.	Schwob et al. (30)
*Casuarina cunninghamiana* (River oak)	*Frankia* sp.	Zimpfer et al. (31)
*Gunnera tinctoria* (Chilean rhubarb)	*Nostoc* sp.	Gioria and Osborne (32)
*Hedera helix* (European ivy)	*Bacillus amyloliquefaciens*	Soares et al. (33)
*Myrica faya* (firetree)	*Frankia* sp.	Burleigh and Dawson; Vitousek et al.; Walker and Vitousek (34 – 36)
Various legumes	Rhizobia (e.g., *Bradyrhizobium* sp.)	La Pierre et al.; Rodríguez-Echeverría et al.; Stepkowski et al. (37 – 39)
*Agrilus mali* (apple buprestid)	*Pantoea* sp. and *Pseudomonas orientalis*	Bozorov et al. (40)
*Agrilus planipennis* (emerald ash borer)	*Streptomyces* sp., *Erwinia* sp., and *Burkholderia cepacia*	Vasanthakumar et al. (41)
*Riptortus pedestris* (a species of broad-headed bug)	*Burkholderia* sp.	Kikuchi et al.; Kikuchi et al. (42, 43)
*Sirex noctilio* (sirex woodwasp)	*Streptomyces* sp. and γ-proteobacteria	Adams et al. (44)
Various coreoid and lygaeoid stinkbugs	*Burkholderia* sp.	Kaltenpoth and Flórez; Kikuchi et al. (45, 46)
Various insects	e.g., gut bacteria	Lu et al. (15)

## A MODEL SYSTEM: LEGUMES, RHIZOBIA, AND RHIZOBIOPHAGES

### Legumes and rhizobia

Soil-dwelling rhizobia infect legume roots, populate intercellular spaces, and stimulate production of specialized organs called nodules (47). In each nodule, a subset of the intercellular rhizobium population is engulfed by host cells and encapsulated within an intracellular symbiosome. There, some rhizobia differentiate into specialized endosymbiotic cells called bacteroids, which reduce atmospheric di-nitrogen (N_2_) in mutualistic exchange for photosynthates (48, 49). Within the nodule, symbiotic rhizobia can reach high cell densities (50 – 52), but once released from senescing nodules, rhizobia must survive the abiotic and biotic stresses of soil (53 – 62).

Free-living rhizobia survive as saprotrophs or persister cells in bulk soil [53, 54, 63, but see reference (64)], which is not penetrated by roots and holds few resources and inhabitants (65, 66). Fortunate cells eventually encounter a rhizosphere (67, 68), the ecologically complex habitat surrounding roots (69 – 72). Rhizobia can proliferate in rhizospheres of both legumes and non-legumes (73, 74), sometimes surviving for years without a legume host (75). The rhizosphere community is rich in both competitors (57, 76 – 82) and natural enemies, such as rhizobiophages (83, 84).

### Rhizobia can facilitate legume invasions

Legumes (Fabaceae) are potent invaders (85). They endanger native plants directly by competition and indirectly by increasing soil nitrogen (N) concentration, which hinders habitat restoration (86, 87) and facilitates non-leguminous invasive plants (88 – 91). A legume species usually partners with particular groups of rhizobia (69, 92 – 96) and obtains greater benefit from familiar, co-evolved rhizobia (37, 97 – 102). However, rhizobia and plants disperse independently (Fig. 1a), with senescing nodules releasing reproductive rhizobia into adjacent soil (103). Rhizobia passively disperse long distances by dust storms (104 – 107) and can also be moved with soil or co-transported with the roots of adult legume hosts (37 – 39, 108 – 116). Nevertheless, rhizobium genotypes are not cosmopolitan and often exhibit a significant biogeographic structure at various spatial scales (37, 117 – 121). Since novel habitats lack familiar rhizobia, establishing legume crops onto new continents requires inoculation with compatible rhizobia (122 – 125). Lacking such deliberate inoculation, range expansion by rhizobium-dependent wild legumes requires that familiar rhizobia either co-disperse or arrive independently (75, 76, 93, 99, 126). How most symbiotic bacteria disperse remains poorly understood (127, 128).

For some legumes, greater soil mineral N can reduce the need for rhizobia (129), but for many legumes, successful invasion depends on the presence or introduction of compatible rhizobia (16, 76, 130 – 132). Despite this dependence, there are multiple cases in which rhizobial symbionts have apparently co-invaded with rhizobium-dependent legumes (37 – 39, 108, 112, 115), with legumes representing almost 10% of the invasive plants recorded for North America (85).

### Rhizobiophages

Surprisingly little is known about rhizobiophage diversity. Early studies classified rhizobiophages by morphology into at least three families (Siphoviridae, Myoviridae, and Podoviridae), all within the order Caudovirales (83, 133). Next-generation sequencing has suddenly increased information about rhizobiophage genomes, revealing a broader taxonomic diversity (84, 134 – 143). However, owing to limited research, the number and diversity of described rhizobiophage genomes available on GenBank comprise only a fraction of the recorded genomes of their rhizobium hosts [e.g., see references (144 – 147)].

The spatial structure of bacteriophage diversity is poorly described in general and known primarily from aquatic ecosystems (148 – 152). It is typically thought that the distribution of a bacteriophage is limited only by the presence of its host, though evidence to support this claim is still missing (153). Bacteriophages can passively move short distances in soil (23) [reviewed in reference (154)], and some phages, either as virions or as prophages, might disperse long distances with wind-borne dust [Fig. 1a (155)] or as stowaways in transported soil. Accordingly, some phages are widely distributed (156 – 158). However, many phage communities are spatially structured (29, 134, 150, 151, 159 – 162), and phage community composition in soil can vary immensely even across small spatial scales [>10 m (163 – 165)]. Communities of rhizobiophages differ strongly among nearby (<10 km distant) legume populations: phages from different agricultural fields of the same host legume rarely showed more than 88% average nucleotide identity (134), and an unpublished analysis of 141 genome sequences of *Bradyrhizobium* spp. from different continents found that all of the 31 detected prophages were unique (J. Van Cauwenberghe, unpublished data). These observations suggest that rhizobiophages disperse poorly over longer distances, but they might nonetheless accompany deliberately applied rhizobium inoculum. Sharma and colleagues (83) detected compatible rhizobiophages in locations where rhizobia were intentionally inoculated onto legumes introduced for afforestation and soil rehabilitation. Often, however, rhizobia being developed for agricultural inoculum are screened for lysogeny (166, 167), and such efforts have been further facilitated by genomic methods (168).

### Phage predation may affect rhizobium success

Mutualism theory predicts that when individual hosts interact with many symbionts, selection favors hosts that can choose the most cooperative symbionts (169 – 171). For example, legumes can constrain infection by compatible but less beneficial rhizobia (70, 92, 172 – 178). However, legumes seldom control which genotypes nodulate them (118, 131, 179 – 182), and legume choice cannot overcome rhizosphere effects (53, 172, 180, 183, 184). For example, crop nodules are rarely occupied by the most effective nitrogen fixers (125, 185, 186) because those genotypes fail to compete in the rhizosphere (54, 186, 187). Instead, the nodulation chances of a rhizobium genotype increases with its cell density in the rhizosphere (184, 188, 189), which means it must compete effectively (125, 190) and survive natural enemies in the rhizosphere (191, 192).

Rhizobiophages are abundant in soils (193 – 195), especially in legume rhizospheres (133, 196, 197), where they can reduce rhizobium nodulation rates and plant growth (198, 199). Phage density is correlated with the decline of free-living (saprophytic) rhizobia in soil (200), and rhizobiophage infection can strongly regulate rhizobium populations (201 – 209). Applying particular rhizobiophages can improve legume crop production by controlling highly competitive rhizobium genotypes that are inefficient N_2_ fixers (202, 203). Rhizobiophages might similarly influence the relative competitive success of native versus introduced rhizobia.

## APPLYING INVASION BIOLOGY THEORIES TO LEGUME-RHIZOBIUM-RHIZOBIOPHAGE SYSTEMS

As with infectious disease epidemics, complex ecological interactions drive the fates of biotic invasions. After an infectious agent is introduced to a host population, the agent can either disappear, lodge as a commensal, or spread. Similarly, depending on the ecological interactions it encounters, an introduced species could immediately disappear, quietly persist with no apparent effect on the native community, or become pestiferous, disrupting the native community, either ecologically or economically or both.

Lytic bacteriophages influence bacterial community composition by causing heavy mortality on specific bacteria (20 – 27, 29, 210, 211). Temperate phages following the lysogenic pathway produce more complex effects. They can confer benefits to their hosts, such as superinfection exclusion (212 – 214) and auxiliary metabolic genes (215, 216), but still turn lethal when they activate their lytic pathway. Thus, bacteriophages might alter the success or failure of bacterial symbionts that can drive the population expansion of introduced eukaryotic hosts. Invasion biology theory helps analyze the many paths along which rhizobiophages could indirectly influence legume invasions (Fig. 1b through g).

Invasion theory (217) proposes mechanisms by which biotic interactions might either facilitate invasion (Fig. 1b, e, and g) or produce “biotic resistance,” i.e., the ability of a native community to resist exotic invasion (Fig. 1c, d, and f). For example, suppressive soils rich in phages that infect *Ralstonia solanacearum* can resist establishment by that plant pathogen (218). Similar mechanisms might be responsible for the aforementioned failure of inoculated rhizobia to competitively occupy either soil (54, 186, 187) or nodule communities (125, 185, 186). We hope this paper stimulates testing of the hypotheses described below.

The earliest invasion biology hypothesis derives from an assumption underlying classical biological control of crop pests (219); i.e., organisms proliferate when introduced in a new range because they arrived without the natural enemies that controlled them in the home range [“enemy escape” or the “enemy release hypothesis” (4, 7, 220 – 222); Fig. 1b]. Eukaryotic hosts commonly proliferate after dispersing over long distances without viral enemies (223). For example, plant species introduced to the U.S. are infected with 24% fewer virus species (224) than in their European home ranges. If eukaryotic hosts arrive and associate with mutualistic bacteria that have dispersed without bacteriophage enemies, the host and bacteria might similarly co-proliferate in the new range. Thus, introduced rhizobia that have escaped compatible rhizobiophages from their home range might outcompete native rhizobia, which remain regulated by their own rhizobiophage enemies, thereby facilitating a legume invasion.

Regardless of whether they escape home range phages, introduced bacteria also encounter “unfamiliar phages”. If evolutionary pressures (e.g., ongoing local adaptation and negative frequency-dependent selection) overcome constraints (e.g., genetic distance and fitness trade-offs), phage host ranges might evolve to encompass previously unfamiliar bacteria (e.g., Fig. 1d through g). Thus, enemy release could be fleeting [e.g., see references (4, 225 – 228)], with the fate of introduced rhizobia depending on how they and native rhizobia interact with phages.

Native phages that can infect introduced rhizobia might hamper co-proliferation of introduced legumes and rhizobia (biotic resistance via “reverse spillover”; Fig. 1d). Alternatively, native phages could facilitate invasion by spilling back onto native bacteria from introduced bacteria [(229, 230) Fig. 1e]. Such “enemy spillback” (231, 232), also called “local pathogen accumulation” (233), could occur if native phages only rarely infect introduced rhizobia (thereby producing little change in the density of introduced rhizobia) but persistently achieve unusually large burst sizes when they do. Because spillback from introduced rhizobia might increase phage density, the phenomenon might be detected by comparing phage abundance in the presence versus absence of introduced rhizobia. Both reverse spillover and enemy spillback can occur in either the presence or the absence of introduced phages (hence the gray lines in Fig. 1d and e)

Non-native phages co-introduced with rhizobia could also either deter or promote co-proliferation and invasion of introduced rhizobia and legumes. Introduced phages might deter invasion simply by continuing to specialize on and regulate co-introduced rhizobia (Fig. 1c). Introduced phages could also deter rhizobium invasion by proliferating more luxuriantly on occasionally infected native rhizobia but most negatively affecting the density of introduced rhizobia (biotic resistance via “subsidized pathogen” [(14) Fig. 1f]. This subsidy of the introduced pathogen could arise if native rhizobia are either more abundant or because they produce comparatively larger burst sizes than introduced rhizobia. We know of no examples of this phenomenon.

Alternatively, introduced phages that infect native bacteria (either rhizobia or other competitors) could facilitate legume-rhizobium invasion by spilling over onto and decimating competing native bacterial communities [“enemy spillover,” a form of “apparent competition” (234 – 236); Fig. 1g]. Although not yet documented for rhizobia, this phenomenon has been observed in other microbial introductions (212, 230, 237, 238). Enemy spillover can occur, e.g., when introduced bacteria carry a prophage, which allows them to outcompete native bacteria that lack resistance to this phage [i.e., phage-mediated allelopathy (239)]. A prophage that is induced in only a few of its lysogenic hosts might continue to replicate lytically on competing susceptible hosts, which could then be eliminated, while protecting its lysogenic hosts via superinfection exclusion (212 – 214). In a recent study simulating bacterial invasions *in vitro*, bacteria dispersing to nearby patches could outcompete native bacteria only when carrying phages to which the latter were susceptible (240).

Thus, regardless of the path along which an invaded community assembles (Fig. 1a), phages can influence invasion by mutualist bacteria. In some scenarios, phages facilitate legume invasion (Fig. 1b, e, and g), whereas in others, they hamper invasion (Fig. 1c, d, and f). Key questions, then, are (i) how often do rhizobia disperse to a new range without their co-evolved rhizobiophages? (ii) how likely is it that rhizobiophage host ranges include or acquire novel rhizobia? (iii) which rhizobia (native or introduced) will be most negatively affected by phages? and (iv) how will rhizobiophage effects on a rhizobium community cascade onto host legume populations?

Whether a phage will affect a novel host bacterium (e.g., spillover) more negatively than its original host (e.g., spillback) depends largely on the relative effectiveness of mechanisms involved in the various stages of infection. These mechanisms include the ability of the phage to attach to each host [e.g., as quantified by adsorption rates (241)], the effectiveness of rhizobium intracellular defense mechanisms, such as restriction-modification systems, CRISPR-Cas systems, abortive infection, and assembly interference [reviewed in references (242 – 244)], or resistance conferred by prophages [i.e. superinfection exclusion (242, 245)], and the phage’s ability to overcome these defenses [reviewed in reference (246)]. How phages will influence outcomes of bacterial competition also depends on population and community-level processes, as discussed below.

## ECO-EVOLUTIONARY FACTORS INFLUENCE HOW RHIZOBIOPHAGES AFFECT LEGUME-RHIZOBIUM INVASION

Rhizobiophages might influence legume invasions by lowering rhizobium density. Indeed, early experiments using single-strain inoculation found that adding rhizobiophages sometimes reduced rhizobium density (205) but not always (206). However, we think rhizobiophages are more likely to influence legume invasions by altering the composition of rhizobium communities (201 – 203, 205 – 207, 209). Accordingly, in experiments creating tripartite microbial communities containing rhizobiophages that infect one of two competing rhizobia, phage-resistant strains often occupy a higher percentage of nodules than phage-sensitive strains (202, 203, 206, 207). For example, a phage specialized on *Bradyrhizobium japonicum* USDA 117 altered the competitive outcome between USDA 117 and *B. japonicum* USDA 110 by reducing in-soil population size and nodule occupancy of its USDA 117 host (202, 203). Such results suggest that rhizobiophages alter apparent competition among rhizobium taxa. Indeed, simple mathematical models of bacterial interactions with lytic phages produce numerical dynamics akin to other predator-prey models (247). However, overlapping temporal scales of evolutionary and numerical dynamics can drive continuing fluctuations following initial community assembly, necessitating a new conceptual framework (24, 28, 248).

Below, we outline some of the decisive factors known to determine how phage communities affect the structure of bacterial communities and suggest, given these principles, which rhizobium community, introduced or native, will be most negatively affected by novel phages. We also consider how these effects might cascade up to affect the invasion potential of a host legume.

### Coevolution

The interdependence of bacterial and phage fitness often produces a co-evolutionary arms race (24, 249, 250): bacteria experience selection for various defensive traits [e.g., alterations to receptors by which bacteriophages attach or mechanisms that recognize and degrade phage DNA or block bacteriophage replication (244, 251)], but bacteriophage populations evolve the ability to use different attachment sites or evade recognition by bacterial hosts (246, 252). A co-evolving partner that fails to counter-adapt quickly faces extinction [i.e., the red queen hypothesis (253)]. Phages typically evolve faster and become locally adapted: more infective on sympatric than allopatric bacteria [e.g., see references (254, 255)]. Thus, naturally occurring phage-bacteria interaction networks usually consist of modules involving local phages adapted to related bacteria (136, 256 – 258) from nearby locations (24, 134, 254, 255, 259). Since the genetic distance between familiar versus recently encountered hosts influences whether a bacteriophage can infect unfamiliar hosts (258, 260) and introduced rhizobia often occupy genetic clusters distinct from native rhizobia (37, 116), phages might not infect unfamiliar rhizobia.

### Specialization

Both phage host range (i.e., the number of types of bacteria a phage can infect and lyse) and the breadth of bacterial resistance (i.e., the number of types of phages a bacterium can resist) are measures of specialization which strongly influence microbial community composition. Generalists, i.e., phages that can infect and lyse more types of bacteria or bacteria that can resist infection by more types of phages, should be more successful than specialists, unless generalization involves trade-offs (261, 262). For example, most ways by which bacteria prevent phage infection are costly to bacterial growth (263 – 266), which limits how many types of phage a bacterium can resist and could also cause bacteria to lose resistance to other phages (267). Similarly, specialist phages might infect and lyse few types of bacteria but obtain larger burst sizes or higher adsorption rates than do generalist phages attacking those same bacteria (268, 269). Thus, the fitness benefits a phage obtains from each bacterium type trade off with the number of bacterial types it can infect and the phylogenetic distances among them [e.g., see references (269 – 274)].

Accordingly, phages are usually specialized within a locality. For example, some rhizobiophages associated with common bean rhizobia were extreme specialists, infecting less than 1% of tested rhizobia (134). However, phages within a community can vary in host range: some rhizobiophages are generalists, infecting more than 90% of local, closely related hosts (134). Phages infecting via more conserved surface receptors may infect a more phylogenetically diverse range of hosts (275, 276). However, even generalist phages are rarely able to infect more than a few taxa and, if so, would only infect certain strains within each taxon (261, 277, 278).

The genetic distance between familiar versus recently encountered bacteria reduces the likelihood a phage can infect such unfamiliar hosts (258, 260). Adapting to new bacteria is most difficult for phages highly specialized on distantly related hosts (258, 260, 271). However, even minor mutations (271, 279, 280) can add new host species or genera (225, 270, 281). Thus, phages might be maladapted only during the initial encounter with novel bacteria (14, 282), e.g., in plant pathogens (232, 283). Native rhizobiophages might adapt to a rapidly expanding population of introduced rhizobia, or introduced rhizobiophages might adapt to the numerically dominant native rhizobia.

### Relative abundance

Frequency-dependent selection favors phages that adopt abundant hosts (284), which causes those hosts to decline. Multiple studies have documented this “kill-the-winner” process (26, 285 – 287). Thus, the relative effect of phages on introduced or native rhizobium communities may depend largely on the initial relative abundances of both communities. Newly introduced rhizobia are likely to be rare (288), which selects for phages that can infect and drive down the abundance of native rhizobia relative to introduced rhizobia. These phages could be either introduced phages, with shifted host ranges, or native phages. Indeed, if introduced phages evolve to infect the more abundant members of the native rhizobium community, they might disrupt the community enough to benefit introduced rhizobia (spillover). As introduced rhizobia proliferate and become invasive, however, selection on phages would reverse. Thus, delayed eco-evolutionary feedback could yield fluctuating-selection dynamics (289).

### Diversity

Rhizobium communities are highly diverse (145, 290, 291), comprising strains with various phage resistance profiles (134), but the diversity of rhizobiophages is still poorly known (136). It is unclear whether and how bacterial diversity predicts how novel phages might structure a host community comprising both familiar and novel bacteria. It is also poorly known how bacterial diversity affects phage evolution [but see reference (274)] and how such evolution could feed back to affect the host community. In kill-the-winner dynamics, “winning” phages can increase bacterial diversity by functioning as keystone predators (292), but a more diverse host community may be more likely to contain bacteria that can survive a greater variety of phages [sampling effect (293)]. Nonetheless, a less diverse bacterial community comprising generalists with relatively broad phage resistance [e.g., with few phage receptors or with effective broad spectrum phage-defense systems (294 – 299)] might still be more resilient to more different phages than is a more diverse community of specialists, each resistant to a different phage (e.g., due to more specialized phage-defense systems).

In general, richer phage communities can better control microbial communities (300, 301), either by including phages with larger or more rapidly expanding host ranges (300 – 303) or by including a greater diversity of specialized phages, each of which attacks different bacterial hosts [sampling effect (293)]. Experiments using phages, either to modify bacterial communities in marine and freshwater systems or to combat pathogenic bacteria in medicine and agriculture (27, 154, 304 – 307), generally find that cocktails of multiple phages provide broader and more durable (i.e., reduced rate at which phage resistance evolves in bacterial hosts) bacterial control than obtained by deploying phages individually (27, 305, 308). However, bacteria are more likely to evolve generalized resistance to a more diverse community of phages (309, 310). For example, Betts et al. (310) found that more diverse phage communities caused selective sweeps of lipopolysaccharide (LPS) synthesis gene mutations, which conferred broad resistance. Nevertheless, introducing even a low-diversity phage community might sufficiently disturb the competitive balance within a bacterial community to compromise its resistance to invasion. Bacterial communities are generally composed of a few dominant genotypes and many rarer genotypes (311, 312), so the decline or eradication of a single dominant genotype via a kill-the-winner process could create a dynamic cascade in which previously rarer genotypes become dominant (26, 287).

### Dispersal affects diversity

The diversity of introduced communities of phages and bacteria depends on their respective large-scale population structure and their introduction histories. Communities and populations usually become genetically depauperate as they disperse over long distances (313, 314), suggesting that native communities of phage and bacteria are likely to be more diverse than those established by a single, small introduction. However, if either phages and/or bacteria have been introduced multiple times from multiple locations, then the introduced communities might be very diverse (313, 315 – 318). Rhizobia co-invading with legume hosts often exhibit evidence of multiple introductions (108, 116), possibly by accompanying more than one species of congeneric legume hosts (39, 116, 319, 320). It would be interesting to compare the diversity of rhizobiophages in such communities with those found in rhizobium communities formed by single introductions.

### Pleiotropic effects of phage resistance in rhizobia

Rhizobiophages can potentially influence legume fate when they select rhizobia with phage resistance traits that share pleiotropic effects with symbiosis or mutualism traits. Phage resistance traits in rhizobia may trade off with their abilities to engage with legumes such as rhizosphere colonization (321), nodulation (322, 323), or nitrogen fixing efficiency (205, 322). Some bacteriophage-resistant mutants of *Bradyrhizobium japonicum* (324) have alterations in cell surface LPSs, which are also common sites of phage attachment (323). Defective LPS prevents nodulation by disabling communication between legumes and rhizobia (190, 325, 326). Alternatively, phage resistance can be associated with more and larger nodules, higher nitrogenase activity (327, 328), and enhanced host nitrogen content (329). Finally, such pleiotropic effects are not always observable (330). Thus, if rhizobia evolve resistance to novel phages, any pleiotropic effects of these traits could either disrupt or improve their cooperation with familiar legumes, depending on the magnitude and direction of pleiotropy.

## CONCLUSIONS AND FUTURE DIRECTIONS

Dependency in rhizobial mutualists appears to be an Achilles’ heel for many invading legumes (16, 37, 93, 112). Thus, legume invasions might be either foiled or promoted by the evolutionary and ecological effects of native and/or co-introduced bacteriophage enemies. The probability of enemy escape is initially determined by the likelihood that the enemy arrives in the new range and subsequently by the adaptive potential of the phages and bacteria, which determines whether introduced rhizobia might be hampered by new enemies or can enjoy the benefits provided by enemy spillover and enemy spillback (Fig. 1).

Our ability to predict the relative probabilities of these various scenarios, both in this system and among other eukaryotes dependent on mutualists infectiously acquired from the environment, is hampered by how little is known about these processes (enemy escape, spillover, biotic resistance via reverse spillover, biotic resistance via subsidized pathogen, and spillback) in this and other bacterium-bacteriophage systems. Progress in this area depends upon identifying bacteriophage communities associated with rhizobia and legumes, and characterizing the ecological and evolutionary interactions among these populations in both native and non-native habitats. Invasions of other eukaryotes dependent on infectiously acquired bacterial mutualists should receive similar attention.
